# Mediation effect of trust on willingness to pay for health insurance among co-operative members in Tanzania

**DOI:** 10.1186/s43093-023-00198-0

**Published:** 2023-04-25

**Authors:** Petro G. Nzowa, Felix A. Nandonde, Somo M. L. Seimu

**Affiliations:** 1 Department of Banking Accounting and Finance (BAF), Faculty of Business and Information Sciences (FBIS), Moshi Co-operative University (MoCU), Moshi, Kilimanjaro Tanzania; 2 Department of Business Management, School of Agricultural Economics and Business Studies (SAEBS), Sokoine University of Agriculture (SUA), Morogoro, Tanzania; 3 Department of Community Development and Gender (CDG), Faculty of Co-Operative and Community Development (FCCD), Moshi Co-operative University (MoCU), Moshi, Kilimanjaro Tanzania

**Keywords:** Health insurance, Willingness to pay, Trust, Co-operatives, Social capital theory, PLS*-*SEM, I13, Q13, H55

## Abstract

This study analysed co-operative members’ willingness to pay (WTP) for health insurance. The social capital theory was adopted to analyse the mediation role of trust issues on other variables determining co-operative members’ WTP for health insurance. A single Contingent Valuation Method (CVM) was used to elicit and estimate the amount co-operative members that were willing to pay for health insurance. The Partial Least Square Structural Equation Modelling (PLS-SEM) was used to analyse variables affecting co-operative members’ WTP for health insurance. Findings indicated that most co-operative members were willing to pay for health insurance. Further, except for price, trust issues fully and partially mediate quality attributes and access criteria, respectively, when it comes to WTP for health insurance. Firm trust is required among co-operators, management, health insurers, and health facilities in order to increase WTP for health insurance among co-operative members.

## Introduction

Globally, health insurance is financed either by the government or individuals or sometimes by both [62]. Conventionally, health insurance financed by the government aims at maximising social welfare and ensuring equity in utilising healthcare services [62, 95]. However, such governments’ goals are constrained by inadequate fiscal space for health and priority gaps [96, 98]. This forces individuals to look for alternative health insurance financing strategies to fill gaps left by the government. Individuals often opt for private health insurance or out-of-pocket expenditure to finance their healthcare needs. However, Balqis-Ali et al. [10] and Sekhri and Savedoff [80] posit that inequalities characterise private health insurance strategies available for individuals and, in most cases, increase exclusions gaps.

As a result of the above handicaps in health insurance financing, more than 50% of the global health expenditure, which amounts to more than USD 7.3 trillion annually, is from out-of-pocket [96]. This is not preferred as high out-of-pocket expenditures result in financial hardship, causing millions of individuals, particularly those in the informal sector, to receive incomplete cures and give up on needed health care. Also, most individuals are pushed into extreme poverty and death [6, 34, 93]. Any government does not desire this situation. Thus, governments worldwide have been trying to implement initiatives to reduce out-of-pocket expenditures and increase the number of individuals with health insurance [6, 41]. One of recommended initiative involves co-opting member-based organisations such as co-operatives to supplement government initiatives to accelerate health insurance financing across populations.

Co-operative institutions have been regarded as a platform  for all aspects of development throughout the globe [40]. These institutions play a significant role in poverty reduction strategies in such aspects as financial inclusion, education and training, agriculture, and social protection, among others [15, 57, 94]. Within this context, co-operative institutions also stem as one of the key players in facilitating and accelerating health insurance coverage for most individuals [41, 42]. The literature, including Seudiband and Amadu [81] and Bastagli [12], shows that the formation and operations of co-operatives accommodate and offer access to social health insurance to individuals neglected and denied by other formal institutions. Hence, countries across the globe are sensitising people to willingly subscribe to health insurance under co-operative institutions to mitigate health challenges and their related costs in an attempt to attain universal health insurance coverage [6, 41, 98]. Yet, the response is still low in most developing economies, most populations are not utilising health insurance services. This necessitates finding why individuals are not willingly paying or subscribing to health insurance initiatives, particularly the voluntary health insurance schemes operated in co-operative institutions.

In Tanzania, the government, through the National Health Insurance Fund (NHIF), created a unique voluntary health insurance scheme for co-operative members, namely “Ushirika Afya” in Kiswahili. The “Ushirika Afya” is a voluntary health insurance scheme designed to serve co-operative members who have no formal and conventional access to health insurance [90]. For other individuals employed in the formal sector, health insurance is mandatory for all workers. Premiums are remitted directly to insurance schemes or companies as employers deduct from their salaries [41]. The “Ushirika Afya” scheme was primarily designed for workers in the agricultural sector to serve members of agricultural and marketing co-operative societies (AMCOS). However, members of other forms of co-operatives can also join the scheme. “Ushirika Afya” acts as a supplementary scheme for co-operative members employed in the formal sector and has a statutory health insurance cover. Therefore, the “Ushirika Afya” scheme has become one of the best platform for health insurance inclusion for individuals statutorily excluded from accessing health insurance. While the scheme plays an essential role in facilitating health insurance, there have been limited empirical investigations showing the extent to which co-operative members and other individuals have utilised such a platform and willingly paid for it.

Health insurance under the “Ushirika Afya” scheme is relatively cheaper than other schemes. Under this scheme, each co-operative member and their spouse voluntarily pay an annual premium of TZS 76,800/- (approximately USD 33) and TZS 50,400/-(approximately USD 22) for children under the age of 21 years. Also, banks in Tanzania, such as NMB Bank PLC, CRDB Bank PLC, and Tanzania Commercial Bank (TCB), offer free-interest health insurance loans to co-operative members to cover the above-stated premium costs. This is an opportunity for most individuals, particularly co-operative members, to increase enrolment in the scheme to expand health insurance coverage in Tanzania. Yet, large segments of the population, including co-operative members, still use out-of-pocket expenditures to address health needs [48, 87]. Statistics show that only 32% of individuals have been accessing health insurance services in the country by 2019, whereby the NHIF covered 8%, while 23% by Community Health Fund (CHF) and the remaining (1%) by private insurers [31, 91, 48]. However, statistics in 2022 indicate that the total Tanzania population covered by health insurance declined to about 15%, of which CHF coverage decreased to about 5.4%, and NHIF remained at 8%. In contrast, private insurers increased coverage to about 2% [91]. This leaves about 85% of Tanzania’s population without health insurance coverage [92]. This has led to challenges such as partial treatment, postponed medical care, and catastrophic health expenditure in case of illness and health eventualities [6, 92, 93]. The question under this situation is whether the opportunities presented in the “Ushirika Afya” scheme have stimulated willingness to pay for health insurance among co-operative members.

Since it is a voluntary scheme, willingness to pay for “Ushirika Afya” depends on the evaluated participation benefits among co-operative members. Also, as the reasons for the low willingness to pay for the mentioned scheme are unknown, recent evidence suggests that the quality of services, access criteria, and pricing are likely to be among the cause [6, 7, 18, 45, 59, 93]. Additionally, Campbell [16], Fenenga et al. [33], and Shan et al. [82] suggest that how individuals trust the actors involved in the health insurance scheme can dictate and control their relationships and abilities in actions concerning willingness to pay for health insurance. So far, however, there is little discussion on whether quality attributes, price and access criteria relating to health insurance services affect co-operative members’ willingness to pay for health insurance, “Ushirika Afya” in particular.

Moreover, the social capital theory postulate that trust issues can intervene and jeopardise individuals’ effective decision-making regarding taking part willingly in various community interventions, particularly health insurance. However, whether trust issues intervene in co-operative members’ willingness to pay for health insurance given the presence of other factors (in this case, price, quality attributes and access criteria) is yet to be studied. In this regard, a need emerges to undertake this study on co-operative members’ willingness to pay for the health insurance scheme designed for them. This study findings are expected to bring new understanding to the scheme designers on areas for improvement and add to the body of knowledge in health insurance operations. This will significantly contribute towards issues that limit the realisation of universal health insurance coverage in Tanzania. Otherwise, the scheme will slowly incur natural death for not achieving the desired outcomes and leave the targeted group uncovered with health insurance. Hence, unimproved health, low level of production among co-operators and subsequently country’s underdevelopment in all aspects.

## Literature review

### Willingness to pay for health insurance

Willingness to pay for health insurance is a proxy measure of cost–benefit trade-offs and hence, a significant factor for variations in using health services across populations [35]. Researchers have investigated contributing factors that pose challenges for willingness to pay for health insurance schemes to most individuals to avoid out-of-pocket expenses for their health needs. Miti et al. [59] and Amani et al. [6] believe that price is among the key factors preventing individuals’ willingness to pay for health insurance. In health insurance literature, price connotes the premium the insured should pay the insurer (insurance company) to be covered [6]. From the basic principle of demand and supply, other things remaining constant, the higher the price, the lower the quantity demanded, and vice versa. Therefore, as the amount of money one needs to pay for insurance premiums increases, the willingness to pay for those individuals decreases [44, 59].

Moreover, studies such as Arkorful et al. [7], Pahlevan Sharif et al. [66], Ebrahim et al. [29], Minyihun et al. [58], Biggeri et al. [13], Lee [53], Dror et al. [26], Panda et al. [67] and Adebayo et al. [1] argue that quality attributes for both insurers and health facilities affect willingness to pay for health insurance among individuals. Quality is considered to be the efforts by health insurers to preserve health and, in case of sickness or injury, to revive health safely and efficiently through the work of health care experts, institutions, and combined delivery systems [26, 53]. In line with that, Biggeri et al. [13] and Adebayo et al. [1] believe that quality should be guaranteed and effectively met in accepted standards for health insurance to be efficient. This means quality is positively related to willingness to pay for health services, particularly health insurance [7, 29, 66]. Impliedly, higher quality of health facilities and insurance providers increases individuals’ willingness to pay for health insurance. Higher quality services increase individuals’ confidence in the service received and health assurance in case of illness.

Likewise, studies by Chiwire et al. [21], Ebrahim et al. [29], Duku et al. [28], and Kusi et al. [52] asserts that access is another factor that is likely to influence an individual’s willingness to pay for health insurance. For Duku et al. [28], access is attributed to equity and easiness among individuals in getting health and health care services as reflected in such aspects as location and distance of health facilities. Similarly, Kusi et al. [52] add that access is reflected in such terms as finding competent healthcare providers willing and able to serve individuals in a near and convenient locality. This is to say, as more individuals are given and open to access to health facilities, they will likely increase their willingness to pay for health insurance [21, 29]. Thus, any deprivation and barriers to access to health facilities for insured and uninsured individuals may result in less willingness to pay for health insurance. This is because access barriers prevent individuals from effectively managing and taking charge of their health and well-being.

However, other studies such as Arkorful et al. [7], Chiwire et al. [21], Miti et al. [59], Amani et al. [6], and Minyihun et al. [58] mentioning just a few assumed uninterrupted relationships between price, quality and access among others as to willingness to pay for health insurance. These studies claimed a linear and direct relationship exists between price, quality, and access as to willingness to pay for health insurance. This claim seems inadequate since the process through which these variables affect willingness to pay for health insurance might be interrupted by other variables. There is a need to introduce an intermediating variable to explain the relationship among these variables better [99]. Thus, this study introduces trust issues as a mediator variable to explain this relationship.

Also, the literature reveals that trust is likely to mediate other factors regarding individuals’ willingness to pay for health insurance [5, 34, 70, 99]. This indicates that trust forms the basis for measuring perceived information, acts, and dealings. Also, Sutter and Kocher [84] claim that trust determines information’s worthiness and truthiness in making and shaping individuals’ decisions. Therefore, it is common to understand that trust guides decisions to pay for health insurance willingly. Similarly, trust between and among individuals, systems, institutions, and the service receiver is the key to successful intervention, particularly in health insurance [36, 55, 77].

A great deal of previous research into health insurance has focused on individuals stating the link between price, quality of services, access criteria, and trust on willingness to pay for it. The extent to which this link holds among co-operative members regarding health insurance, “Ushirika Afya” in particular is still unclear. To establish such clarity, this study chains to this area of research by analysing co-operative members’ willingness to pay for health insurance.

### Theoretical framework of the study

#### The social capital theory

This study is governed by the social capital theory (SCT). Proponents of the SCT argue that elements of social connection and ties govern interactions and provide generative benefits among individuals, groups, and community members [22, 73, 74, 78, 83]. Such interactions can be affected by such elements as the level of trust, solidarity, and reciprocity amongst individuals within the group or community [61, 74]. These elements dictate bonding and regulate one’s capabilities for decision-making and participation in social issues for equitable enjoyment of expected benefits [25, 30, 32, 33, 50]. Further, such elements can emanate as individual attributes [43, 72] or external forces (group attributes) [47], or as both individual and group attributes [71] when comes to affecting and influencing the decision. This study confines itself to one central SCT element, trust. Trust is analysed to see how it dictates and regulates bonding and capabilities as to willingness to pay for health insurance among co-operative members. The adoption of the trust element is based on Putnam’s [73] argument that social capital is fundamentally the degree of trust between individuals that facilitates their actions and collaborations for mutual gain.

In Tanzania, co-operatives have gone through different apogees. At a time, co-operatives were very strong, and several initiatives through these institutions were successful. Also, there was a time when co-operatives lost their direction due to various reasons such as malpractices and embezzlement among leaders. This is when co-operative members were marginalised and lost trust and hope. However, in the 1980s, co-operative revived and gained its lost glory. Following that revival, co-operatives have been assigned responsibilities and are used to speed economic development and improve members’ welfare. One of the signed responsibilities is facilitating health insurance delivery through various schemes to its members who do not access it in conventional ways.

Based on the dynamics that co-operatives have gone through, the assumption is that members of co-operatives are likely to lose trust in their institutions and among themselves. In that regard, using social capital theory with an element of trust is appropriate for this study. So we think for the co-operatives to be a vehicle to accelerate health insurance through these schemes, social capital is significant. The assumption is that if individuals trust each other and their institution, they are likely to increase their willingness to pay for health insurance through schemes such as “Ushirika Afya”. Further studies in insurance affirm that social capital elements, trust in particular, increase willingness to pay and enrolment in health insurance given that other factors such as price, quality, access, and other benefits are in order [16, 33, 37, 101]. Hence, this study uses SCT to explain how trust issues will likely influence and control co-operative members’ relationships concerning health insurance. Also, SCT explains abilities regarding willingness to pay for health insurance, particularly* “*Ushirika Afya” and other health schemes meant for co-operative members.

### Hypotheses development and conceptual framework

#### Price

The literature pinpoints price as a proxy measure of an individual’s ability to pay for financial services, particularly health insurance. They argue that price affects the ability of these individuals to join and renew and triggers dropout in many voluntary health insurance schemes [44, 59]. Also, price is an exclusion driver for the majority to willingly pay for health insurance across countries [44, 59, 63, 67]. Price influences and affects decisions for willingness to pay for health insurance by allowing individuals to analyse the perceived cost–benefit of the service. Thus, it is likely that whenever the price is in favour, the willingness to pay will be higher and vice versa. However, this is only known to the general public and individuals. Whether the same is likely to happen for co-operative members when it comes to paying for health insurance willingly is yet to be studied. Hence, this study hypothesises the following:

##### H_1_

Price has a negative relationship with willingness to pay for health insurance among co-operative members.

#### Quality

Literature indicates that the quality of the insurer and the health service provider determines individuals’ willingness to pay for health insurance. Few to mention, the willingness to pay for micro-health insurance among rural and poor people [26], community-based health insurance [75], co-operatives health insurance [3, 4], and mutual health organisations [88]. Willingness to pay is stimulated when people are satisfied with the service quality [2, 7]. It creates a sense of confidence about the diagnosis and treatments individuals receive.

Also, quality attributes are related to the dropout or continued membership in terms of premium payments or contributions to voluntary health insurance schemes [6, 19, 26, 58, 60, 79]. Here, quality attributes make individuals compare the amount and costs paid for health insurance and actual services received to justify if it is a fair deed. Any variations between the expected and actual quality of services form the basis for terminating the health insurance contract. Yet, of all this empirical evidence, little is known about whether quality attributes influence willingness to pay for health insurance among co-operative members. Thus, the following hypothesis is proposed:

##### H_2_

Quality of the service by insurers is positively related to willingness to pay for health insurance among co-operative members.

#### Access

It is also advocated that the insured’s access to the service location determines willingness to pay for insurance services among individuals [2, 28, 46]. Unlimited admittance to various health centres, forms and types is likely to influence willingness to pay for health insurance [6, 28, 52, 58]. Inclusion or exclusion criteria on access to either public/government or private hospitals and specialised clinics where the insured are accepted for treatments influence decisions on willingness to pay for health insurance [6, 58, 59, 86]. This is because it increases confidence and guarantees the insured to get services without any exclusion criteria in all areas. Thus, any barriers to access, such as long distance to the health facility, denial of some services for the insured, and restrictions on the frequency of using insurance cards per day, are expected to reduce the willingness to pay for health insurance among individuals. However, to what extent and direction access affects willingness to pay for health insurance among co-operative members is still opaque to fill. It is therefore hypothesised that:

##### H_3_

Access criteria are negatively related to co-operative members’ willingness to pay for health insurance.

#### Trust

Studies reveal that individuals do not trust and are not willingly paying for the existing health insurance systems. They fear losing their money in terms of annual insurance premiums, especially when they do not get sick [54]. Further, they hesitate to contribute such premiums to an organisation and individuals unfamiliar with them and have no relationship with them due to trust issues [79]. Lack of trust among individuals in the institutions or schemes offering insurance services results in low demand or minute response in contributions because premiums are paid in advance and benefits are received in the future [54, 76, 99]. Nonetheless, firm trust in service delivery increases individuals’ response to willingly pay and participate in health insurance regardless of its prevailing conditions [11, 49].

To revive trust and increase willingness to pay and participate in health insurance for those who are statutorily negated, governments and other key players have thought of and adopted co-operatives as a channel to deliver formal health insurance [100]. It is assumed that co-operatives’ formation process and operations resemble the traditional way of helping each other in case of contingencies. Co-operative principles, values, and practices give a sense of trust to one another and any initiative that can be brought into it from outside. Nonetheless, it is the best source for financing health insurance and health care for household members [94, 100]. More precisely, trust is anticipated to positively affect the willingness to pay for health insurance when attributed to other factors like quality, price, and access to insurance services [5, 34]. Probably, the higher the trust, the more the willingness to pay for health insurance [8, 29]. However, to the researcher’s knowledge, limited studies have been conducted to justify whether trust issues have anything to do with the willingness to pay for health insurance among co-operative members. This has led to the formulation of the hypothesis that:

##### H_4_

Trust issues have a negative relationship with willingness to pay for health insurance among co-operative members

#### Hypothesised mediation effect of trust on willingness to pay

Trust issues are expected to intervene in the relationships between price, quality and access and willingness to pay for health insurance among co-operative members. By starting with price, in most cases, the insured pay a certain premium when trust in the insurer prevails. The prevailing trust among the insured in the health insurance schemes or companies makes them continue to pay for health insurance willingly [99]. However, the ability to pay insurance prices may be affected by the variability in individuals’ (in this case, co-operative members) trust in the insurers and management of co-operatives, that in turn, affect willingness to pay for health insurance [5, 8]. The assumption is that regardless of the co-operative members’ ability to pay the premium set, if they mistrust “Ushirika Afya” operations in various dimensions such as leadership, fund management and alike, they will not willingly pay for the scheme. Therefore, we hypothesise that,

##### H_5_

Trust issues mediate the effect of price on co-operative members’ willingness to pay for health insurance.

As for quality, dependable quality attributes increase individuals’ trust in the services they receive, increasing their willingness to pay for the health insurance scheme [29, 64]. Quality raises individuals’ trust in aspects such as preserving and reviving health safely and efficiently through the available delivery systems accepting health insurance [53]. However, regardless of the quality of services, if members have trust issues, their desire and willingness to pay for health insurance are also affected [29, 69, 101]. Based on the SCT affirmation, co-operative members’ decision to willingly pay for the “Ushirika Afya” is expected not only on the scheme’s quality but also on their trust, which determines bonding among each other and the management of the scheme. Thus, this study hypothesises that,

##### H_6_

Trust issues mediate the effect of quality on willingness to pay for health insurance among co-operative members.

Despite the insured’s convenient access to health facilities determining their willingness to pay for insurance [28, 52, 59], they always prefer accessing health facilities that they trust [49, 79]. Overall, unlimited access to health facilities among the insured increases their confidence and guarantees them to get needed and preferred health services [59]. This increases individuals’ willingness to pay for health insurance [58]. However, there is a need to assess whether trust issues intervene in co-operative members’ willingness to pay for “Ushirika Afya” health insurance in given conditions for access. Therefore, it is hypothesised that,

##### H_7_

Trust issues mediate the effect of access criteria on co-operative members’ willingness to pay for health insurance.

Based on the above hypotheses and by showing the relationship among the variables, the study is conceptualised as under: -

## Methodology

### Study design

Quantitative method research was adopted to measure the relationship between the independent variables (price, quality, access and trust) and the dependent variable (willingness to pay) mediated by trust. A cross-sectional survey design was used in this study. The design enabled the collection and analysis of data on the variations in independent variables to the dependent variable at a single point in time. Kilimanjaro and Arusha regions were selected to give out respondents for the study representing other regions of Tanzania where health insurance has been introduced in co-operatives. The area was selected because of its outstanding history of co-operatives movements and practices. Arumeru and Moshi Districts were selected from the selected regions because co-operatives suiting this study’s demands were available. The co-operatives selected were Aranga AMCOS, Mrimbo Uuwo AMCOS, Marangu East AMCOS, Kikarola SACCOS, and Mamba South AMCOS. The co-operatives selected in the area comprise diverse members with different abilities relating to pricing, quality, access, and trust regarding willingness to pay for health insurance. Moreover, the selected co-operatives are currently or have been incorporated into health insurance operations by health insurance providers.

### Data collection instruments

The questionnaires were developed using a five-point Likert scale ranging from strongly disagree (1) to strongly agree (5) to collect opinions on the influence of independent variables (price, quality, and access) on the dependent variable (willingness to pay for health insurance (“Ushirika Afya”)) when mediated by trust issues. The study adopted the Five-point Likert scale because the respondents involved in the study were not considerably exposed to the Likert scale measurement. Hence, the scale enabled them to make fine distinctions among variables parameters, increasing the potential for information gain [51, 68]. The development of questionnaires for this study was inspired and adapted from other previous studies. Then, it was refined and customised to suit the requirements of this study. The items for the price construct were adopted from the work of Sweeney and Soutar [85]. Moreover, the items for the *trust issues* construct were adapted from Boateng and Narteh [14], while items for the *access criteria* construct were modified from the study of Liu et al. [54]. Likewise, Lee [53] and Urbach et al. [89] inspired the formation of items for the *quality attributes* construct regarding health insurance services.

### Data collection and analysis procedures

Before the data collection, a pilot study was conducted to test respondents’ understanding and relevance of questions. The pilot involved a sample of 50 respondents, who accounted for 10% of the total sample size for the study. After pilot testing, alterations were made to the questions to reflect and fit respondents understanding so as to be effective in collecting sufficient and relevant information. Such modifications included changing the terms and language that were irrelevant to the respondents’ level of understanding. Also, some variables were dropped because they were irrelevant to the co-operative members’ context.

A total of 550 responses were randomly collected from co-operative members to form the base of analysis in the study between August 2019 and December 2020. Data collection took a longer time than usual because of the COVID-19 pandemic. Due to the pandemic, local government authorities and co-operative leaders hesitated to permit data collection, and co-operative members were uncertain about participating in the study.

Hair et al. [39] recommend addressing issues of missing data and sceptical responses prior data analysis stage. They further state that in partial least square structural equation modelling (PLS-SEM), a 10 times rule of thumb, that is, 10 times the largest number of formative indicators used to measure a single construct, can be used to determine the appropriate sample size for the analysis in the study. Since a single formative construct with the largest number of indicators had 4 indicators, 40 respondents were adequate for analysis in this study. However, from the 550 collected responses, 53 responses were dropped after checking for missing values and suspicious responses. Finally, 497 questionnaires were fit, sufficient, and good enough for quality structural equation model analysis, as Wolf et al. [97] and Comrey and Lee [23] recommended.

A single Contingent Valuation Method (CVM) was used to elicit and estimate the amount co-operative members that were willing to pay for the “Ushirika Afya” scheme. Respondents were given four ranges to choose from about the amount they would pay for health insurance. CVM is a widely and commonly adopted survey-based technique to estimate individuals’ WTP for a product not conventionally traded in the marketplace [24, 27]. In the insurance sector, CVM involves surveying the target populations’ responses to the maximum price they would be willing to pay for hypothetical insurance products after being enlightened about their benefits [27]. The technique was adopted because it allowed the researcher to get direct and explicit financial risk trade-offs of the respondents about the nature, depth, and monetary implications of the amounts on the table [9, 56] for the “Ushirika Afya*”* insurance product.

Moreover, PLS-SEM is adopted to analyse variables that affect co-operative members’ willingness to pay for health insurance. The model is used to determine the extent of variables relationships and structural model association in this study, as commended by Hair et al. [38]. PLS-SEM is adopted because it allows a distribution-free variance and gives maximum explained variance [65]. Further, PLS-SEM is suggested when evaluating formatively measured complex models with limiting effects on both observed and latent indicators, as it is for this study [39, 65].

## Findings and discussion

This segment presents the findings and discussion of the study. The first section discusses the descriptive findings of the study. A discussion of the measurement model, the structural model, and hypothesis testing follows. In the sections below, the phrase “willingness to pay for health insurance” is used to imply “willingness to pay for “Ushirika Afya” Scheme and other health insurance schemes/packages targeting co-operative members.”

### Descriptive findings

Out of the 497 respondents who correctly and duly completed questionnaires, findings indicate that, on average, the number of family members was 5, while on average, each family had 3 dependants. The findings also indicated that the average age of the respondents was 50 years. Furthermore, findings indicated that 69% of the respondents were males while 31% were females. Findings also indicated that 84.7% of the respondents were married, 9.9% were single, 0.8% were divorced, and 4.6% were widowed. Additionally, it was found that 18.3% of respondents were government employees, 19.9% were private sector employees, 61.2% were self-employed, and 0.6% were unemployed. Out of 497 respondents, 492 were willing to pay for health insurance, while 5 were not, as shown in Table 1.

**Table 1:** Descriptive findings

Variable	Willingness to pay for health insurance		
	Yes (*n* = 492; 98.99%)		No (*n* = 5; 1.01%)
Pay current price	Yes (*n* = 430;87.4%	No (*n* = 62; 12.6%)	
Gender	N(497)		
Male	342 (69%)		
Female	155 (31%)		
Marital status			
Married	421 (84.7%)		
Others	76 (15.3%)		
Employment/Occupation Status			
Government employee	94 (18.3%)		
Private sector employee	99 (19.9%)		
Self-employed	304 (61.2%)		
Average age	50 years		
Average household size	5		
Average number of dependants in the household	3		

Also, this study intended to analyse if co-operative members were willing to pay above or below the prices offered by health insurance funds or companies. Currently, the NHIF charges an annual premium of TZS 76,800/- for each co-operative member and their dependents (Spouse and parents) and TZS 50,400/- for children below 18 years. They all receive the same service coverage in accredited health facilities. On the other hand, private health insurance companies charge annual premiums ranging from TZS 30,000/- (approximately USD 13) to TZS 220,000/- (approximately USD 94) per individual, with varied healthcare service cover based on the premium paid. Most members who were willing to pay for health insurance in this study (430 = 87.4%) (Table 1) were willing to pay the exact and relatively above the prevailing prices. However, this was for the members benefiting or integrated with the NHIF in the “Ushirika Afya*”* Scheme. Nonetheless, about 12.6% (62) of the respondents, particularly those insured by private health insurance companies, were reluctant to continue paying the current price and were willing to pay relatively below the prevailing prices.

Based on the “arm’s length transactions” tradition, the findings indicate that most members receive relatively fair service for the price paid. Thus, additional prices should reflect further improvements in the quality of the services and more access to the facilities and services offered by respective schemes.

### Formative measurement model

In PLS-SEM, either a reflective or formative measurement model can be adopted. This study adopted a formative measurement model to analyse the mediation effect of trust on willingness to pay for health insurance. The model was formatively measured because each indicator explicitly captured the construct’s domain [39]. Thus, convergent validity, collinearity between indicators, significance, and relevance of outer weights were to be determined. In assessing convergent validity, a correlation of above 0.70 in the formative indicator construct is appropriate [20, 39]. As can be seen in Table 2, redundancy analyses of the formatively measured constructs Price, Quality, Access, and Trust generated scores of 0.917, 0.761, 0.829, and 0.783, respectively. Thus, all of the constructs conform to convergent validity.

**Table 2:** Convergent validity and collinearity statistics

Formative constructs	Convergent validity	Formative indicator	VIF
Price	0.917	WTPHinsPrc1	1.194
		WTPHinsPrc2	1.004
		WTPHinsPrc3	1.193
Quality attributes	0.761	WTPHinsQlty1	1.063
		WTPHinsQlty2	1.220
		WTPHinsQlty3	1.241
		WTPHinsQlty4	1.176
Access criteria	0.829	WTPHinsAcs1	1.057
		WTPHinsAcs2	1.163
		WTPHinsAcs3	1.185
Trust issues	0.783	CoopMembTrsPHins1	1.034
		CoopMembTrsPHins2	1.064
		CoopMembTrsPHins3	1.101
		CoopMembTrsPHins4	1.047
WTP	0.945	WTP1	1.047
		WTP2	1.025

Hair et al. [39] recommend looking at the variance inflation factor (VIF) as a proxy measure of collinearity among indicators. A VIF of less than 5 for an indicator indicates no potential collinearity among indicators [39]. The findings for the collinearity test are shown in Table 2, where all indicators have a VIF of less than 5. Thus, there is no potential threat of collinearity among formative constructs that might affect the estimation and evaluation of the structural model on willingness to pay for health insurance.

Next was the evaluation of the indicators’ outer weight, outer loading significance, and relevance. The assessment intends to measure indicators’ exclusive significance and relevance in specifying contents and explaining the constructs [39]. Table 3 shows formatively measured constructs findings indicating variables’ estimates for outer weights, outer loadings, *t* values, and *p* values, together with confidence intervals obtained by the percentile method (BCa). The rule of thumb is that formative indicators’ outer weights should be significant at *ρ* < 0.05, or the formative indicators’ outer loading value > 0.5 so as to be kept for analysis, otherwise removed [17, 39].

**Table 3:** Formative constructs outer weights significance testing result

Relationship	Outer weights	Outer loadings	T statistics	97.5% BCa C.I	*P* values	Significance
Trst1-> TRUST	0.557	0.609	5.198	0.344, 0.770	0.000	Yes
Trst2-> TRUST	0.160	0.529	1.916	− 0.010, 0.321	0.055	No
Trst3-> TRUST	0.359	0.572	5.237	0.234, 0.498	0.000	Yes
Trst3-> TRUST	0.550	0.649	7.033	0.414, 0.715	0.000	Yes
WTP1- > WTP	0.089	0.509	1.415	− 0.033, 0.217	0.157	No
WTP2-> WTP	− 0.237	− 0.634	2.234	− 0.533, − 0.017	0.013	Yes
Acs1-> ACC	0.283	0.467	3.362	0.095, 0.430	0.001	Yes
Acs2-> ACC	− 0.062	0.518	0.845	− 0.211, 0.076	0.398	No
Acs3-> ACC	0.917	0.955	17.410	0.816, 1.021	0.000	Yes
Prc1-> PR	0.022	− 0.522	0.460	− 0.279, 0.113	0.645	No
Prc2—> PR	0.512	0.518	1.248	− 0.850, 0.970	0.212	No
Prc3-> PR	− 0.185	− 0.189	1.998	− 0.650, − 0.162	0.046	Yes
Qlty1-> QLTY_	0.131	0.281	2.003	− 0.006, 0.257	0.045	Yes
Qlty2-> QLTY_	0.397	0.718	5.489	0.254, 0.535	0.000	Yes
Qlty3-> QLTY_	0.630	0.862	8.431	0.489, 0.779	0.000	Yes
Qlty4-> QLTY_	0.225	0.544	3.711	0.103, 0.341	0.000	Yes

In Table 3 above, the outer weights estimates for the formative indicators have *ρ* < 0.05 or the outer loading value > 0.5. Therefore, with all the indicators having met the threshold, hence are kept in the model since they significantly inform the key constructs of the study, that is, price, quality attributes, access criteria, and trust issues as far as willingness to pay for health insurance is concerned.

### Structural model measurement

**Fig. 1:**
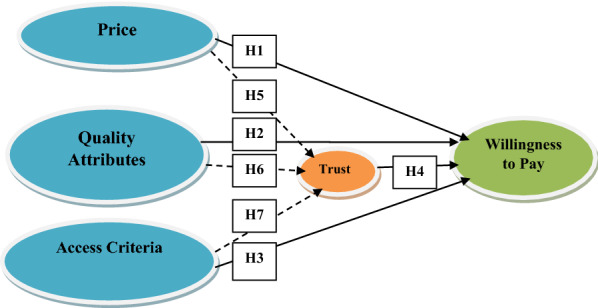
Conceptual framework of this study

Structural model measurement involves the analysis of the total effects of each exogenous construct price, quality attributes, access criteria, and trust issues to exhibit its relationships to the endogenous formative construct, which is the willingness to pay for health insurance. Further, specific indirect effects were measured to assess the meditation role of trust issues on the willingness to pay for health insurance among co-operative members. Tables 4 and 5 show the results of hypothesis testing after the PLS-SEM algorithm analyses of the interactions stipulated in the conceptual model (Fig. 1).

**Table 4:** Total effects

Relationship	Hypotheses	Β	Standard deviation	T statistics	*P* values
PR-> TRUST		− 0.245	0.225	1.091	0.276
PR-> WTP	H_1_	− 0.122	0.053	2.311	0.021
QLTY_-> TRUST		0.302	0.083	3.644	0.000
QLTY_-> WTP	H_2_	0.118	0.062	1.895	0.058
ACC-> TRUST		0.245	0.051	4.794	0.000
ACC-> WTP	H_3_	0.136	0.053	2.552	0.011
TRUST-> WTP	H_4_	− 0.233	0.072	3.228	0.001

**Table 5:** Specific indirect effects

Relationship	Hypotheses	Β	Standard deviation	T statistics	*P* values
QLTY_-> TRUST-> WTP	H5	− 0.070	0.035	2.021	0.043
PR-> TRUST-> WTP	H6	0.057	0.052	1.100	0.271
ACC-> TRUST-> WTP	H7	− 0.057	0.021	2.699	0.007

Analysis was performed to assess the role of price on willingness to pay for health insurance among co-operative members. The findings (Table 4) revealed that the price of health insurance in terms of premium paid significantly negatively impacts willingness to pay (H_1_: *β* =  − 0.122 *t* = 2.311 *ρ* = 0.021). Hence, H_1_ is accepted; that is, the price has a negative relationship with willingness to pay for health insurance among co-operative members. From the findings, as health insurance prices increase by 1 unit, co-operative members’ willingness to pay decreases by more than 12% and vice versa. These findings are in line with the studies by Miti et al. [59], Amani et al. [6], and Jofre-Bonet and Kamara [44], who found that a higher premium for health insurance leads to a lower willingness to pay. Since price acts as a proxy measure of co-operative members’ ability to pay, its increase jeopardises their willingness to pay for health insurance. Also, a price increase reduces the number of members who renew their membership, triggering dropout from health insurance schemes among co-operative members. However, the free interest health insurance loan given by banks (NMB, CRDB, and TCB) reduced the burden of finding the money to cover the premium costs and stimulated willingness to pay for “Ushiraka Afya” among co-operative members.

Also, this study assessed the role of health insurance quality attributes on willingness to pay for health insurance among co-operatives members. Surprisingly, the findings (Table 4) revealed that the quality attributes of the health insurance provider and health facilities have no significant impact on willingness to pay (H_2_: *β* = 0.118, *t* = 1.895, *ρ* = 0.058). Hence, we fail to accept H_2_ as quality attributes does not positively relate to the willingness to pay for health insurance among co-operative members. This shows that the relationship between quality and willingness to pay is insignificant. This means co-operative members’ willingness to pay for health insurance remains unchanged as to variations in the quality attributes of health insurance services. This finding contradicts previous studies’ findings (e.g. [3, 4, 6 and 19] that quality influences willingness to pay and continued membership in health insurance schemes. The implication of this study findings is that co-operative members does not consider quality attributes as one of the stimulants for their willingness to pay for health insurance. Possibly, the current quality attributes of the health insurance and health facilities reflect what is expected of the amount or costs paid for insurance cover. However, these findings might indicate that something beyond quality attributes (e.g. Trust) is needed to stimulate and increase their willingness to pay for health insurance.

Further, this study analysed the influence of access criteria on willingness to pay for health insurance among members of co-operatives. The findings (Table 4) revealed that access criteria significantly positively impact willingness to pay (H_3_: *β* = 0.136, *t* = 2.552, *ρ* = 0.011). This shows that the relationship between access criteria and willingness to pay for health insurance is positive. Hence, we fail to accept H_3_ that access criteria are negatively related to willingness to pay for health insurance among co-operative members. As insurers and health facilities vary access criteria by 1 unit, willingness to pay varies by 13.6% in response to such variations in access criteria. This finding concurs with studies such as Amani et al. [6], Minyihun et al. [58], Duku et al. [28], and Kusi et al. [52]. They claimed that unrestricted entry and convenient access to several types and forms of health facilities would likely influence willingness to pay for health insurance. This implies that willingness to pay for health insurance increases when co-operative members have more access without exclusions criteria to health facilities. This is to say the willingness to pay for health insurance rises when the insured co-operative members have confidence and guaranteed access to nearby public/government or private hospitals and specialised clinics without restriction and limited frequency of using insurance cards per day.

On the other hand, analysis was performed to assess the sole role of trust issues on willingness to pay for health insurance. The findings (Table 4) revealed that trust issues significantly negatively impact willingness to pay (H_4_: *β* =  − 0.233, *t* = 3.228, *ρ* = 0.001). This shows that the relationship between trust issues and willingness to pay for health insurance is negative. When trust issues increase among co-operative members to the actors of health insurance by 1 unit, willingness to pay decreases by 23.3%, and vice versa. Thus, we fail to reject H_4_ as the findings indicate that trust issues negatively affect co-operative members’ willingness to pay for health insurance. A negative relationship between trust issues and willingness to pay for health insurance was also reported by Zein et al. [99], Liu et al. [54], Fenenga et al. [33] and Shan et al. [82]. Based on these findings, we believe that trust predicts and can mediate other variables towards willingness to pay for health insurance among co-operative members. When co-operative members trust health insurance providers, health facilities and their management, they will be more willing to pay for health insurance. Contrary to that, any negative variation in the degree of trust among co-operative members to the insurers and health facilities can impair patronage and sustainability of the health insurance scheme.

### Mediation analysis

Having analysed the significance of total effects in the model, the specific indirect effects were then analysed to test the mediation role of trust issues on willingness to pay for health insurance among co-operative members.

The analysis was performed to assess the mediating role of trust issues in the linkage between price and willingness to pay for health insurance. Despite the findings (Table 4) revealing the total effect of price on willingness to pay to be significant, the inclusion of mediating variable (Trust issues) in analysing the impact of price on willingness to pay became insignificant (Table 5) (H_6_: *β* = 0.057, *t* = 1.100, *ρ* = 0.271). This shows that trust issues do not mediate the relationship between price and willingness to pay because the total effect and specific indirect effect were insignificant. Thus, we fail to accept H_6_. This means trust issues do not mediate the effect of price on willingness to pay for health insurance among co-operative members. These findings contradict Alhassan [5] and Attia and Price [8] who concludes that despite the ability to pay for the existing price, variability in members’ trust affects their willingness to pay for health insurance. This study findings reflect that willingness to pay for health insurance is not affected by how co-operative members trust the insurers and health facilities but rather by their ability to pay the premiums for the service. The findings might imply that if co-operative members cannot afford to pay the premium for the health insurance, their trust issues cannot be related to their willingness to pay for it. Moreover, these findings can be attributed to the free of interest health insurance loans given to co-operative members to cover the cost of premium. Since the loan is given without interest and it is paid by the bank direct to the health insurance provider, they might not subject such payments with their trust issues on the respective schemes.

However, the relationship between quality and willingness to pay in the presence of a mediator reveals different findings. When mediation analysis was performed to assess the mediating role of trust issues in the linkage between quality attributes and willingness to pay for health insurance, the findings were significant (H_5_: *β* =  − 0.070, *t* = 2.021, *ρ* = 0.043). With the inclusion of mediating variable (Trust issues), the impacts of quality on willingness to pay became significant; that is, trust issues negatively and significantly mediates the effect of quality on willingness to pay for health insurance among co-operative members. Hence, we fail to reject H_5_. This shows trust issues fully mediate the relationship between quality and willingness to pay for health insurance because the total effect (Table 4) was insignificant, while the specific indirect effect (Table 5) became significant. This finding implies that co-operative members’ satisfaction with quality attributes of the health insurance provider and health facilities alone cannot serve as a determinant to increase their willingness to pay for health insurance. This implies that an increased willingness to pay for health insurance among co-operative members depends on how they enjoy and appreciate the quality of health insurance services and how they trust the schemes. Furthermore, these findings shows that for the co-operative members to willingly pay for health insurance of a given quality attribute, trust issues concerning the health facilities, staff, and medical equipment used to serve them should be minimal. These findings concur with other studies on quality influence on the willingness and intention to pay and use health insurance. Such studies are Arkorful et al. [7], Ebrahim et al. [29], and Phe Goursat and Pellerano [69]. They found that in the presence of trust, quality factors positively influence individuals’ willingness to use and pay for health insurance.

This study also analysed the mediating role of trust issues in the linkage between access criteria and willingness to pay for health insurance. The findings (Table 5) revealed that the total effect of access criteria on willingness to pay was significant (Table 4) (*β* = 0.136, *t* = 2.552, *ρ* = 0.011). With the inclusion of mediating variable (Trust issues), the impact of access on willingness to pay also became significant (Table 4) (H_7_: *β* = 0.193, *t* = 3.504, *ρ* = 0.000). This shows that trust issues partially mediate the relationship between access criteria and willingness to pay for health insurance because both the total and specific indirect effects become significant. Hence, H_7_ is accepted as trust issues mediate the effect of access on willingness to pay for health insurance among co-operative members. Such a relationship was also reported by scholars, including Ebrahim et al. [29], Alhassan [5], Fenny et al. [34], and Attia and Price [8]. Thus, favourable access criteria alone do not fully guarantee an increased willingness to pay for health insurance. Co-operative members also need to trust individuals or institutions offering health insurance and health services for them to increase their willingness to pay. Whenever co-operative members incline trust issues with the operations of the health insurance scheme or management of their co-operatives, their willingness to pay is likely to decline regardless of the access criteria that are in place. This indicates that despite having unrestricted or biased access, they should not fear being serviced by unfamiliar individuals or institutions for them to pay for health insurance willingly. On the other hand, given the access criteria, when co-operative members fully trust the health insurance system and co-operative management, their willingness to pay for health insurance is more likely to increase.

## Theoretical implications, conclusion, and recommendations

### Theoretical implications of the study

The findings of this study hypothesise two interesting theoretical inferences for scholars. First, even though social capital theory hypothetically emphasises trust to mediate the relationship between price and willingness to pay, this study’s findings contradict with the theory as trust issues had no role to the co-operative members’ context. However, price negatively and significantly influences co-operative members’ willingness to pay for health insurance. The second implication of the study is the absence of a direct relationship between quality attributes and willingness to pay for health insurance in the structural model. At the same time, mediation analysis incorporating trust issues revealed a relationship between quality attributes and willingness to pay for health insurance. This supports the social capital theory as trust issues among co-operative members proved to dictate and regulate bonding and capabilities as to willingness to pay for health insurance. The implication is that the degree of trust among and between co-operative members facilitates their actions and collaborations as to their willingness to pay for health insurance. Thus, whenever individual co-operative members or groups of members incline a huge trust in health insurance actors, they will expect health insurance services to be of reasonable quality with minimum barriers to access to pay for it at a given price willingly.

### Conclusion and recommendations

Overall, co-operative members are willing to pay for health insurance at the current price and even at a relatively higher price, given that they trust the scheme and barriers to access are moderate. On the contrary, in the absence of trust as the mediator, quality attributes do not influence willingness to pay for health insurance among co-operative members. Also, the study findings indicate that except for price, trust issues fully and partially mediate quality attributes and access criteria, respectively, as to willingness to pay for health insurance among co-operative members.

For this, willingness to pay for health insurance is affected by trust issues relating to health insurance quality attributes and access criteria among co-operative members. Hence, for an increased willingness to pay for health insurance among co-operative members, firm trust is needed among co-operators, management, health insurers, and health facilities. Also, for this to work, co-operative leaders in corroboration with health insurance providers and health facilities must strive to ensure appropriate and acceptable quality of health insurance packages and services of the health facilities accredited to serve the insured.

Similarly, reduced barriers, guaranteed access to nearby health facilities, and the frequency of insurance card usage per day were also related to an increased willingness to pay for health insurance among co-operative members. Thus, health insurance operators must devise mechanisms in place that intend to create more room for insured co-operative members and other individuals to access health services smoothly and conveniently. Implementing the above will contribute significantly to the initiatives towards realising universal health insurance coverage in Tanzania.

### Limitations of the study

Despite the study’s significant contribution to practical and theoretical aspects regarding willingness to pay for health insurance, the base for analysis resides only on co-operative members. Thus, one should generalise this study’s findings cautiously as the idea of willingness to pay for health insurance cuts across diverse populations. Yet, the stated limitation does not nullify the significance of this study findings and its contribution to the literature on health insurance. The study is a base for future empirical studies investigating the willingness to pay for health insurance in Tanzania.

## Data Availability

Data are available upon request.

## Abbreviations

AMCOS: Agricultural and marketing co-operative societies
BCa: Bias corrected and accelerated
CHF: Community health fund
COVID-19: Coronavirus disease 2019
CVM: Contingent valuation method
ILO: International labour organisation
NHIF: National health insurance fund
PLC: Public limited company
PLS-SEM: Partial least square structural equation modelling
SACCOS: Savings and credits co-operative societies
SCT: Social capital theory
TCB: Tanzania commercial bank
TZS: Tanzania shillings
URT: United Republic of Tanzania
USD: United States dollar
VIF: Variance inflation factor
WHO: World Health Organization
